# Empirical research on international environmental migration: a systematic review

**DOI:** 10.1007/s11111-014-0210-7

**Published:** 2014-02-22

**Authors:** Reiko Obokata, Luisa Veronis, Robert McLeman

**Affiliations:** 1 Department of Geography, University of Ottawa, 60 University, Room 017, Ottawa, ON K1N 6N5 Canada; 2 Department of Geography and Environmental Studies, Wilfrid Laurier University, 75 University Avenue West, Waterloo, ON N2L 3C5 Canada

**Keywords:** Environmental migration, Environmental refugees, International migration, Environment-migration nexus, Systematic literature review

## Abstract

This paper presents the findings of a systematic review of scholarly publications that report empirical findings from studies of environmentally-related international migration. There exists a small, but growing accumulation of empirical studies that consider environmentally-linked migration that spans international borders. These studies provide useful evidence for scholars and policymakers in understanding how environmental factors interact with political, economic and social factors to influence migration behavior and outcomes that are specific to international movements of people, in highlighting promising future research directions, and in raising important considerations for international policymaking. Our review identifies countries of migrant origin and destination that have so far been the subject of empirical research, the environmental factors believed to have influenced these migrations, the interactions of environmental and non-environmental factors as well as the role of context in influencing migration behavior, and the types of methods used by researchers. In reporting our findings, we identify the strengths and challenges associated with the main empirical approaches, highlight significant gaps and future opportunities for empirical work, and contribute to advancing understanding of environmental influences on international migration more generally. Specifically, we propose an exploratory framework to take into account the role of context in shaping environmental migration across borders, including the dynamic and complex interactions between environmental and non-environmental factors at a range of scales.

**Electronic supplementary material:**

The online version of this article (doi:10.1007/s11111-014-0210-7) contains supplementary material, which is available to authorized users.

## Introduction

It has been nearly 30 years since the term “environmental refugees” came into regular use (El-Hinnawi 1985) and 20 years since ecologist Norman Myers issued his first of several warnings in scholarly journals that the twenty-first century would see hundreds of thousands of people flee their homes for causes directly or indirectly attributable to the environment (Myers 1993, 1997, 2002). Having since recognized the problematic legal and conceptual limitations of the term “refugee” in this context (McAdam 2013) (although it continues to be used widely in the popular media), researchers and informed policymakers have been devoting increasing attention to the potential causes of environmental migration more generally, its legal and governance implications, the future risks posed by climate change, and the role of migration within broader processes of adaptation. Reviewers of the field (e.g., Piguet 2010; Bates 2002; Gemenne 2011; McLeman 2011, 2014; Warner 2010, among others) have observed that discussions about environmental migration are often normative in nature and have called for greater empirical research. This need is increasing, for the subject has moved well beyond the academic sphere. Policy discussions about the security risks of anthropogenic climate change, including population displacements and migration, have reached the United Nations Security Council on two separate occasions (UN Security Council 2011), and it is becoming increasingly likely that adaptation planning and funding under the UN Framework Convention on Climate Change will eventually include the development of strategies for resettling populations from highly vulnerable areas like small island states (for discussion, see Biermann and Boas 2012; Gibb and Ford 2012; McLeman 2014). As pressure grows to generate new policies and programs to respond to migration arising from existing environmental risks such as droughts, floods, land degradation, and storms, as well as new risks related to climate change and mean sea-level rise, the empirical evidence that already exists becomes of increasing value to decision makers.

Most migration, whether its causal influences are environmental in nature or otherwise, occurs within national boundaries (Gill 2010; McLeman 2014; Samers 2010; Weeks 2008). Consistent with this trend, much of the available empirical research focuses on internal migration following events such as the Great Plains Dust Bowl of the 1930s, Ethiopian droughts of the 1980s, flooding and dam construction along China’s Yangtze River, and Hurricane Katrina (e.g. McLeman 2006; Meze-Hausken 2000; Yan and Qian 2004; Fussell et al. 2010). While there is much in that body of literature that can inform decisions about environmental migration across borders as well, international migration has its own particular, additional dynamics owing to the structural influences of states’ policies and regulations regarding migration and citizenship (and the resulting constraints these may place on migration and mobility), and the larger governance structures and processes that make one state potentially more desirable as a place to live or work as compared with others. There exists a smaller, but growing accumulation of empirical studies that consider environmentally-linked migration that spans international borders, and it is these that particularly interest us for the present article and our broader research on the implications for policy and governance of international migration. Although there is certainly a need for many more, these existing studies provide useful evidence for scholars and policymakers in understanding how environmental factors interact with political, economic and social factors to influence migration behavior and outcomes that are specific to international movements of people, in highlighting promising future research directions, and in raising important considerations for international policymaking.

With these considerations in mind, we undertook to create and analyze an inventory of peer-reviewed, scholarly publications that report empirical findings from studies of environmentally-related international migration. In doing so, we sought to identify countries of migrant origin and destination that have so far been the subject of empirical research, the environmental factors believed to have influenced these migrations, the interactions of environmental and non-environmental factors (particularly economic factors) as well as the role of context (including macro-level structures and micro-level factors such as demographics) in influencing migration behavior, and the types of methods used by researchers. Our study extends and builds upon previous work by Piguet (2010), who reviewed then-existing methodological approaches with an emphasis on the role of environmental factors in migration that is already occurring and McLeman (2013), who reviewed developments in modelling of climate-related migration. In reporting our findings we identify the strengths and challenges associated with the main empirical approaches, highlight significant gaps and future opportunities for empirical work, and contribute to advancing our understanding of environmental influences on international migration more generally.

## Methodology

We used an approach adapted from Berrang-Ford et al. (2011) to generate systematically an inventory of English-language peer-reviewed articles that examine environmental factors and international migration published as of mid-2013. The first key criterion was that these articles must deal specifically with international (as opposed to, or in addition to, internal) environmental migration. For the purposes of this study, we took “environmental migration” to mean migration where environmental events, conditions, changes in those conditions, and/or the physical impacts of such changes have been explicitly identified by the author(s) of the article as having an influence on the occurrence or outcome of that migration event, a definition modified from McLeman (2014, p. 10). In this definition, environmental factors need not be the sole cause of the migration event. Using various combinations of terms (shown in Table 1), a keyword search was done in ISI Web of Science, which has been shown elsewhere to be particularly useful for this type of research (Berrang-Ford et al. 2011; McLeman 2011; Jasco 2005). This search generated a list of 2,718 documents whose titles and abstracts were reviewed to eliminate those that did not meet the criteria for this study, leaving us with 72 articles that were included in our inventory for initial detailed analysis. Excluded documents included non-peer-reviewed publications (e.g., book reviews, commentaries, editorials, policy documents, and conference reports); articles that did not pertain to human migration (some matches addressed non-human migration); and matches that were duplicates by the same author(s). The reference lists of the 72 included articles were then sampled to identify additional articles fitting our criteria but that were not captured by the original search terms. This same resampling process was repeated until no new references were identified, providing a final inventory of 92 articles meeting the original criteria.

**Table 1:** Keywords used in the systematic review of English language peer-reviewed articles on environmental change and international migration

Environment	International	Migration
“Climate change” Deforestation Desertification Disaster Drought El Niño Environment* Fire Flood* “Heat wave” Hurricane Landslide “Natural hazard” “Sea-level rise” Tornado Tsunami	International*	Migrant[s] Refugee[s] Displace*

An asterisk following a word indicates a Boolean search for variants with the same root (e.g., flood, floods, flooding)

We used a standardized questionnaire to analyze each of these articles and recorded the origins of each article (i.e., year published, authors, journal, discipline); the type of article (theoretical review, legal/policy, literature review, empirical research, etc.); the environmental factors discussed (drought, sea-level rise, etc.); countries of migrant origin and destination; types of migration (e.g., refugee, labor migration); and asked several qualitative questions of each article regarding the linkages between environmental and other drivers of migration, and the nature of migration outcomes. A copy of this questionnaire is provided in the Supplementary Materials.

Our second key criterion was to single out examples of empirical research, by which we mean studies where the authors collected primary data, or applied new analytical models to existing data. This focus reduced the inventory further, to a total of 31 articles. For these articles, we developed additional analytical questions, including the types of methodologies (quantitative, qualitative, or mixed) and methods (modeling, surveys, interviews) used; the authors’ treatment of non-environmental factors such as class, gender, age, and livelihood; and the relationship between migrant agency and structural constraints on migration. The remainder of this article summarizes our findings and offers a discussion of significant observations and opportunities for future research.

## Results

### Migrant sending and receiving countries

**Fig. 1:**
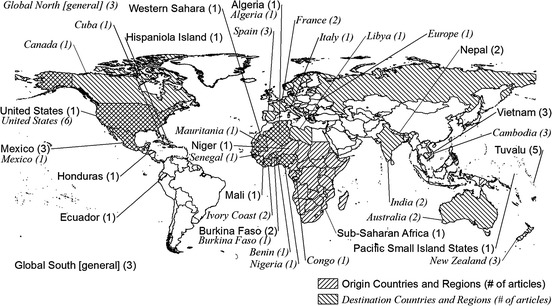
Origin and destination countries and regions examined in the articles under review

Of the 31 empirical articles in the inventory, all but five looked at migration from specific countries or regions (see Fig. 1). The remainder (Bettini 2013; McNamara 2007; Marchiori and Schumacher 2011; Reuveny and Moore 2009; Rowlands 2004) considered international environmental migration in a more general sense or through broad North–South analyses. Countries in Africa figured in the largest number of studies (seven articles), followed by Pacific Islands (six articles), Central America and the Caribbean (six articles), Asia (five articles), and South America (two articles) (Table 2). Five articles (Dun 2011; Gila et al. 2011; Radel et al. 2010; Shen and Binns 2012; Shen and Gemenne 2011) reported empirical information on both a migrant source country and the destination country. Within all countries and regions, the most common study areas were environmentally marginal areas and/or low-income rural areas where residents depend closely on natural resources for subsistence.

**Table 2:** Origins, destinations, and environmental drivers of international migrants in the articles under review

Author(s)	Origin	Current international destination(s)	Environmental drivers investigated
Afifi (2011)	Niger	Benin, Burkina Faso, Congo, Libya, Nigeria, Europe	Drought, land degradation, access to resources (Lake Chad, Niger river), deforestation
Alscher (2011)	Hispaniola Island	Cross-border on island, United States, Canada, France, Spain	Natural disasters, flooding, deforestation, land degradation
Bettini (2013)	None specified (“South”)	None specified (“North”)	None specified
Dun (2011)	Vietnam	Cambodia	Flooding, natural disasters
Farbotko (2005)	Tuvalu	Australia	None specified
Farbotko and Lazrus (2012)	Tuvalu	Australia, New Zealand	Sea-level rise
Feng et al. (2010)	Mexico	United States	Agricultural productivity
Findley (1994)	Mali	France, Senegal, Ivory Coast	Drought
Gila et al. (2011)	Western Sahara and Algeria	Algeria, Spain, Italy, Mauritania, Cuba	Drought, access to resources (grazing/farming land), water shortages; locusts
Gray (2009, 2010)	Ecuador (Southern Andes)	Spain, United States	Land quality, agricultural productivity
Henry et al. (2004a, b)	Burkina Faso (five rural regions and three cities)	Cote D’Ivoire	Drought, land degradation
Kniveton et al. (2011)	Burkina Faso	None specified	Drought
Marchiori and Schumacher (2011)	None specified (“South”)	None specified (“North”)	Climate change
Marchiori et al. (2012)	Sub-Saharan Africa	None specified	Rainfall anomalies, temperature anomalies
Massey et al. (2010)	Nepal	India	Access to resources (firewood), agricultural productivity
McNamara (2007)	None specified	None specified	None specified
McNamara and Gibson (2009)	Pacific Small Island States	None specified	Sea-level rise
Mortreux and Barnett (2009)	Tuvalu	None specified	Sea-level rise
Nawrotzki et al. (2013)	Mexico	United States	Drought
Radel et al. (2010)	Mexico	United States	Drought, natural disasters
Reuveny and Moore (2009)	None specified	None specified	Natural disasters, land degradation, access to resources (crop land)
Rowlands (2004)	None specified (“South”)	None specified (“North”)	Deforestation
Shen and Binns (2012)	Tuvalu	New Zealand	Sea-level rise
Shen and Gemenne (2011)	Tuvalu	New Zealand	Sea-level rise, flooding, land degradation, waste disposal
Shrestha and Bhandari (2007)	Nepal	India	Access to resources (firewood)
Sunil et al. (2007)	United States	Mexico	Climate, natural amenities (pull factors)
Warner (2010)	Vietnam	Cambodia	Flooding, sea-level rise, natural disasters
Warner et al. (2010)	Vietnam	Cambodia	Flooding, sea-level rise
Wrathall (2012)	Honduras (Garifuna villages)	United States	Natural disasters, flooding

Studies did not need to meet all specified attributes shown above to be included in the inventory, so long as the key criteria for inclusion were met

### Evidence that environmental factors influence international migration

A significant question we asked was whether the empirical evidence bears out the starting assumption that environmental factors can influence international migration. Of the 31 empirical articles reviewed, 23 found some evidence of environmental factors influencing migration across international borders, while five others (Farbotko and Lazrus 2012; McNamara and Gibson 2009; Mortreux and Barnett 2009; Shen and Binns 2012; Shen and Gemenne 2011) that looked for evidence did not find it. These five studies examine small island states in the Pacific, a region which is widely believed will be a future site of large-scale population displacements due to sea-level rise. While researchers have found significant levels of international migration from highly vulnerable Pacific states like Tuvalu, at present, little of it can be directly attributed to environmental factors as compared with economic factors or traditions of mobility. The three remaining articles (Bettini 2013; McNamara 2007; Farbotko 2005) use empirical methods to critically discuss international environmental migration, but do not investigate particular case studies with regard to origins and/or destinations.

A number of qualifications should be made to support these results. First, the majority of the reviewed studies focus specifically on rural populations that participate in subsistence activities. While subsistence livelihoods make people highly susceptible to the negative impacts of environmental events and change and may therefore predispose them to becoming potential migrants (see Hunter 2005), subsistence means they may not have the necessary capital assets to undertake anything more than short-distance migration during times of environmental stress (McLeman and Smit 2006). When international migration does ensue for subsistence households, it often leads to destinations in close physical and cultural proximity to their own (e.g., Afifi 2011; Alscher 2011; Dun 2011). In Shrestha and Bhandari’s (2007) study of rural migration patterns in Nepal, the authors conclude, “the transaction cost of migration between domestic and international destination may not be substantial because of the open border, cultural homogeneity, and the short distance to the Indian border from the Chitwan Valley” (p. 35). In the cases in which internal migration was investigated alongside international migration (e.g., Dun 2011; Findley 1994; Gray 2009; Henry et al. 2004a), the number of internal migrants was typically found to be greater than the number of international migrants. In addition, international migration is shown in these articles to be highly dependent on a number of other non-environmental factors at various scales, an issue that will be addressed in-depth below.

### Environmental drivers

The most frequently studied environmental phenomena in these articles are (in decreasing order of significance): drought, land degradation, flooding, access to contextually significant natural resources, sea-level rise, natural disasters, agricultural productivity and deforestation (see Table 3). To clarify, by contextually significant resources, we refer to resources and subsistence activities that are context specific such as firewood availability in Nepal’s Chitwan Valley (Shrestha and Bhandari 2007; Massey et al. 2010), grazing and farming land for Sahrawi pastoralists in Western Sahara and Algeria (Gila et al. 2011), crop land availability in Reuveny and Moore’s (2009) North–South model, and the shrinking and polluting of Lake Chad and the Niger River in Niger (Afifi 2011).

**Table 3:** Types of environmental factors examined in the articles reviewed

Environmental factors	Articles where mentioned
Drought	Afifi (2011), Findley (1994), Gila et al. (2011), Henry et al. (2004a, b), Kniveton et al. (2011), Nawrotzki et al. (2013), Radel et al. (2010);
Land degradation	Afifi (2011), Alscher (2011), Henry et al. (2004a, b), Reuveny and Moore (2009), Shen and Gemenne (2011)
Flooding	Alscher (2011), Dun (2011), Warner (2010), Warner et al. (2010), Wrathall (2012)
Access to contextually specific natural resources (e.g., firewood)	Afifi (2011), Gila et al. (2011), Massey et al. (2010), Reuveny and Moore (2009), Shrestha and Bhandari (2007)
Sea-level rise	Farbotko and Lazrus (2012), McNamara and Gibson (2009), Mortreux and Barnett (2009), Shen and Binns (2012), Shen and Gemenne (2011)
Natural disasters	Alscher (2011), Dun (2011), Radel et al. (2010), Reuveny and Moore (2009), Wrathall (2012)
Agricultural productivity	Feng et al. (2010), Gray (2009, 2010), Massey et al. (2010)
Deforestation	Afifi (2011), Alscher (2011), Rowlands (2004)

Some tentative connections can be made between certain types of environmental factors and certain types of migration (e.g., drought with internal, short distance, temporary migration in Africa—e.g., Findley 1994; Henry et al. 2004a, b). However, the relatively small number of empirical studies, the wide array of study sites, and the diversity of methods used, make it difficult to draw anything more than tentative conclusions as to which environmental phenomena have the greatest tendency to stimulate migration. Moreover, the empirical evidence shows that the same environmental factor may have differing effects on migration from one country to another. The seeming incoherence of findings is strongly linked to a number of contextual and methodological differences between the articles—both issues which we discuss in detail below. Finally, it cannot be overstated that the articles reviewed demonstrate that environmental influences rarely, if ever, act as a sole “push” factor of migration, meaning that political, economic, social, and demographic factors constantly interact with direct connections to whether, or how far, a person may migrate in times of environmental stress.

### Interactions of environmental and non-environmental influences on migration

A recurrent theme in the articles under review is the complex relationship between environmental and economic and political factors in migration causality. Studies that found environmental factors to influence international migration also found that the environment is rarely the sole driver. Multi-causality is particularly apparent in studies where authors made efforts to investigate migrant and non-migrant perceptions within the broader context of the country or region of origin. For instance, in some qualitative studies, it was common for research participants to state their reasons for migration to be economic and for the authors to subsequently trace these economic motivations to changes in natural capital assets related to participants’ livelihoods (e.g., Afifi 2011; Alscher 2011; Wrathall 2012). In eight of the case studies (Afifi 2011; Alscher 2011; Feng et al. 2010; Findley 1994; Gray 2009, 2010; Massey et al. 2010; Nawrotzki et al. 2013; Shrestha and Bhandari 2007), slow-onset environmental changes, particularly drought, are a factor influencing individuals’ decisions to migrate because of the economic consequences experienced at the household level (e.g., crop loss). This process whereby environmental deterioration exacerbates economic instability has been dubbed “environmentally induced economic migration” by Afifi (2011), who observed in Niger that people appear to migrate primarily due to economic problems, but that these problems are closely linked to environmental degradation.

In other studies, the key ongoing factors driving international migration are political or economic (e.g., poverty, personal debt, a weak state, or a lack of infrastructure or state programs), with rapid-onset environmental changes causing surges in migration. An example is Wrathall’s (2012) case study of Honduras, where Hurricane Mitch destroyed homes and productive capital, leaving many residents unable to pay back loans or continue their livelihoods. Out-migration was a common response, especially among young men and the more well-off members of the community, creating a consequent decline in the communities’ human capital (consistent with more general adaptive migration processes described in McLeman and Smit 2006). Noteworthy is that some of the participants in Wrathall’s study explicitly described their migration motivations as being economic in nature despite Mitch being an obvious root cause. The following quote from one of his interviews highlights the blurry distinction between environmental and economic motivations for migration when disasters strike: “my son’s migration…isn’t directly tied to Mitch, but rather because he can’t earn anything here. Yes. We lost everything with Mitch, and yes, the economy was destroyed here, but in the end, he left to find a job” (p. 591). In this case, while the environmental event appears a decisive factor in triggering migration, the broader economic and political context—poverty, lack of government support, employment activities, etc.—ultimately seems to determine whether a person or population opts to stay or leave.

In the case of Hispaniola Island, Alscher (2011) observed a cascading causality effect where multiple environmental factors—tropical storms, flooding, deforestation, and soil erosion—have interacted with current and historical socioeconomic and political factors to stimulate migration. While environmental factors are seen to play a role in migration decisions, the absence of government support is seen to be the real catalyst for the need to migrate. In other words, in the face of environmental hardships, Alscher’s interviewees indicate that few adaptation options are available other than migration.

Interviews conducted by Wrathall (2012) and Alscher (2011) draw attention to the dynamic interplay between agency and structure in migration decisions, i.e., the tension between “analysing the individual and small-group agency of the actors of migration on the one hand and the historical–structural forces shaping migration on the other hand—notably the geography of wealth and power” (King 2012; p. 137). We would argue that simplistic structure–agency polarizations do not correspond to the lived realties related by many of the interviewees in these articles who speak to a fluid interaction between their own decision-making and the ways in which they are able to adapt to environmental conditions due to broader sociopolitical constraints in their regions of origin. In sum, the empirical evidence shows complex linkages between environmental and non-environmental factors and strongly suggests that migration outcomes are context specific.

### The role of context

In the articles under examination, context plays a key role in explaining the differential impacts of environmental factors on international migration. In fact, the articles reveal that context is itself a complex factor and includes different types of processes at different scales. We wish to articulate that while our review below distinguishes between environmental and non-environmental context and takes a scalar approach to discuss different types of context, we do so for analytical purposes. The authors reviewed here do not make these distinctions, and we certainly lean toward critical approaches that stress the dynamic interactions between these different dimensions and scales of context.

#### Environmental context

Taken together, the empirical studies reveal that the specificity of environmental context is important in determining not only the presence and effects of particular environmental phenomena in particular locales, but also in determining which environmental features are so crucial to people’s subsistence that their diminishing presence may influence migration (i.e., firewood in Nepal). Additionally, the effects of a specific phenomenon, such as drought, should be examined contextually. For example, Afifi (2011) shows that there is some particularity about the local impact of drought in northern Niger, where the shrinking of Lake Chad has had profound effects on the out-migration of fishers, farmers and those whose businesses rely on the presence of those communities. There, the environmental context refers to a historical dependence on Lake Chad as a vital resource which has been adversely affected by drought and increased anthropogenic demand. Afifi’s interviewees’ discussions of the relationship between migration and the shrinking of Lake Chad—rather than drought more broadly—provided a particularly nuanced example of links between environment and migration.

At the same time, other articles suggest that examining environmental context should not imply the adoption of only a narrow local or regional analysis. McNamara and Gibson’s (2009) study shows the importance of “scaling up” and assessing the relationship between environmental context at the local level (in this case, the issue of sea-level rise), and how this factor is related to larger global inequities (including resource extraction, emissions, and climate change effects). Furthermore, Farbotko (2005) and Farbotko and Lazrus (2012) challenge the ways the local scale is imagined in terms of marginality and vulnerability.

#### The non-environmental context

As suggested earlier, the articles demonstrate that environmental context is tightly intertwined with a range of factors that make up the non-environmental context and that their complex interactions play a key role in determining the adaptive capacity of a region and social group.

First, some authors situate their case studies within a macro-historical perspective and provide information about the colonial and to some extent postcolonial contexts (Afifi 2011; Alscher 2011; Gila et al. 2011; Shen and Binns 2012), which assists in showing the relationship between these historical processes and the migration processes of particular countries, regions, and people today. In Hispaniola Island, Niger, and Western Sahara, the colonial history seems to have lessened local adaptive capacity due to high levels of external debt, cash-cropping, and imposed sedentarism, respectively. On the other hand, colonial ties may be contributing to Tuvalu’s adaptive capacity through trading relationships, aid money, and contribution to Tuvaluans’ historical and present mobility (Shen and Binns 2012). For some communities in the articles reviewed, mobility has historically been a part of their coping strategies or livelihoods, whether through nomadism for the Sahrawi people (Gila et al. 2011), seasonal migrations (Afifi 2011; Findley 1994; Henry 2004a, b), and mobile occupations such as fishing (Farbotko and Lazrus 2012), which represent traditional forms of adaptation to environmental change. However, macro-historical factors may play a role in hindering the ability to use mobility as a coping strategy such as in Gila et al.’s (2011) discussion of Sahrawi refugees. Fears about a new wave of climate migration should be assessed within this context where sedentarism is not necessarily the norm (Farbotko and Lazrus 2012), even though it should be recognized that contemporary forms of environmental change may lead to different or new types of mobility compared to these traditional forms.

Next, the articles under review also show that this broader context must be examined alongside other aspects of context at finer scales. Some authors (Afifi 2011; Alscher 2011; Dun 2011; Henry et al. 2004a, b; Kniveton et al. 2011; Massey et al. 2010) provide information on the political and economic structures of the nations and regions within which their case studies are situated. The presence or absence of institutions, state programs, and policies impact the local level of adaptive capacity, and thus determine whether or how migration takes place. Nevertheless, the empirical evidence suggests that the outcomes in terms of migration vary greatly depending on the nature of the programmes (e.g., improve local adaptive capacity vs. resettlement), the context of implementation (prevention vs. adaptation, pre- or post-disaster), and the groups targeted (e.g., landowners or landless people).

In Alscher’s (2011) study on Hispaniola Island, participants explain that migration is their only option, due, in part, to a lack of government programs or subsidies to support farmers affected by environmental change. In this case, migration is a form of adaptation when there is no state support. On the other hand, four case studies mention or describe the role of government-sponsored resettlement plans, with differing effects on reducing vulnerability (Dun 2011; Warner 2010; Warner et al. 2010 same case study; Henry et al. 2004a, b; Massey et al. 2010; Wrathall 2012). Dun’s case study examines migration in the Mekong Delta in relation to a state-implemented resettlement program designed as a disaster risk reduction strategy. However, Dun points to an important class dynamic that while resettlement “reduces the physical vulnerability of exposure…it can increase the social and economic vulnerability of resettled communities” (p. e208) because the relocate tend to be the poorest and landless. Those who are wealthier and also own land away from the riverbank appear to be more resilient and able to migrate on their own terms without the same fear of losing social networks and employment opportunities. International migrants may not have received the government assistance needed to stay within Vietnam, with many of Dun’s international interviewees indicating that they saw no other option other than to migrate to Cambodia. Gila et al. (2011) describe how international migrations out of Algerian refugee camps act like a state-sponsored temporary resettlement plan where the most vulnerable Sahrawi refugees (children, those with health problems) are sent to Spain for “environmental holidays” to escape the harsh conditions of the camps. This state initiative seems to be a positive intervention in the lives of the refugees and acts as a form of adaptation to environmental stress. There are also examples of adaptation programs being implemented after migration has occurred, as in Afifi’s (2011) case of Niger, where an environmental restoration program was initiated by the government as an incentive to keep young men within the country.

Finally, the empirical studies also show that community, household, and individual context (e.g., class, gender, ethnicity/race, age, and human, social and economic capital) are important factors in determining whether migration occurs, and if so, what type of migration may be undertaken (internal or international, temporary or permanent, etc.). However, the articles reviewed suggest that the influence of these smaller scale factors varies depending on the more macro-scale environmental and non-environmental contextual factors discussed above. We also note that the scale of analysis varies across the articles under review depending on the theoretical and methodological approaches adopted. For example, some studies including those taking a New Economics of Labour Migration approach use the household as the level of analysis (Massey et al. 2010; Gray 2009, 2010; Radel et al. 2010; Shrestha and Bhandari 2007; Nawrotzki et al. 2013).

The importance of undertaking a multi-scalar analysis is underscored by the number of studies with conflicting findings across different case studies. One example is the effect of land ownership. Research in Ecuador suggests it may provide a source of capital for potential international migrants (Gray 2009, 2010), but in rural Nepal, land ownership appears to be associated with lower levels of participation in international migration (Massey et al. 2010). The influence of education also appears to be context specific, with research in Burkina Faso suggesting educational attainment is more positively associated with internal migration (Henry et al. 2004a, b), but in Nepal and Ecuador, education has a greater positive association with international migration (Massey et al. 2010; Gray 2009). In the case of the Dominican Republic, international migrants tend to have lower educational attainment (Alscher 2011). Conflicting findings can also be found with regard to the same environmental problem: for example, while drought has been shown to have a positive effect on migration from rural Mexico to the US (Feng et al. 2010; Nawrotzki et al. 2013), drought had a negative effect on mobility in Mali during the 1980s (Findley 1994). As reviewers, we cannot with confidence explain why there are such conflicting findings across different case studies, except to point the necessity of situating the role of education or land ownership, for example, within a global and national context, as well as to differential community and household-level dynamics across the case studies.

Studies that examine community and household-level international migration behavior have identified a wide range of potentially influential factors. Social capital (particularly familial connections with other migrants) and previous migration experience facilitate migration from Honduras (Wrathall 2012), Mali (Findley 1994), and Nepal (Massey et al. 2010), for example. In Nepal and West Africa, migration can become an embedded component of rural households’ wider livelihood diversification strategies, with larger family size making migration more possible (e.g., Afifi 2011; Shrestha and Bhandari 2007; see also Mertz et al. 2009). Studies also show that the propensity to migrate can be associated with particular ethnicities or social classes, although these are not independent variables, but are in turn closely linked with economic, social and cultural processes. For example, in Niger, Tuaregs from the north of the country are relatively wealthier than other, larger ethnic groups due to their high levels of participation in extractive industries, and will undertake migration to Europe as a matter of prestige (Afifi 2011). In southern Niger, where farming is a main livelihood, migrants are more likely to migrate internally or to neighboring African countries, with environmental degradation having a greater causal influence. In Nepal, environmental factors have the greatest influence on the movements of lower-caste members of the community, with high-caste Hindus being relatively untouched by most measures of environmental stress other than time taken to collect fodder (Massey et al. 2010).

Gender can play a particularly complex role in the process of international environmental migration. Most studies find that long-distance migration is undertaken by younger men and men who are heads of households (Findley 1994; Gray 2009; Henry et al. 2004a, b; Radel et al. 2010; Wrathall 2012). But this is not always the case. For example, in the Dominican Republic, Alscher (2011) describes environmental migration as a chain event (rural–urban-international) that leads women to seek work in domestic services overseas. Nevertheless, authors also note the gendered impacts that high rates of male out-migration can create as well as the challenges for those who are left behind. This is the case in Niger where Afifi (2011) explains:

only women, elderly and children stay, a fact that has a negative impact on environmental restoration…Women undertake this work indeed…but they miss the physical support of the young men who leave. In some cases, teams of women are doing all the work to restore the environment, which is not sufficient, especially since they have other priorities, such as taking care of the children and the elderly. Furthermore, if the husbands do not send sufficient money, the women have to work to get food instead. (p. e112)

Alscher (2011) describes a similar situation in Haiti, where even in remittance-receiving households, children drop out of school to assist with subsistence farming. In other cases, whole communities can be affected by the migration of more well-off community members whose departures may lead to less local investment, fewer employment opportunities, a smaller customer base for commercial enterprises, and an aging population (Wrathall 2012). In other words, migration can indirectly bring about a range of side effects from further environmental deterioration to economic and social instability.

Finally, context also plays a role, albeit indirectly, when it comes to methodological questions such as what data are available (depending on the countries or environmental factors), what units of analysis are chosen, and what type of methodological approach is undertaken. We address these issues in the section below.

### Methods

Researchers have used a variety of research approaches and methods in their empirical work, which can be loosely organized according to three categories: quantitative methods and modelling (14 articles in total); qualitative research (six articles); and studies that combine a mixture of the former two methods (11 articles) (Table 4). Each method has its own strengths and weaknesses in terms of explanatory potential. Several useful reviews of developments in methodologies already exist (e.g., McLeman 2013; Piguet 2010; Warner 2011); here, we highlight several points that stood out when reviewing our inventory.

**Table 4:** Articles by type of research methods

Quantitative methods and modelling	Qualitative	Mixed methods
Feng et al. (2010)	Bettini (2013)	Afifi (2011)*
Findley (1994)	Farbotko (2005)	Alscher (2011)*
Gray (2009)	Farbotko and Lazrus (2012)	Dun (2011)*
Gray (2010)	McNamara (2007)	Gila et al. (2011)*
Henry et al. (2004a)	McNamara and Gibson (2009)	Radel et al. (2010)
Henry et al. (2004b)	Mortreux and Barnett (2009)	Shen and Binns (2012)
Kniveton et al. (2011)		Shen and Gemenne (2011)*
Marchiori and Schumacher (2011)		Sunil et al. (2007)
Marchiori et al. (2012)		Warner (2010)*
Massey et al. (2010)		Warner et al. (2010)*
Nawrotzki et al. (2013)		Wrathall (2012)
Reuveny and Moore (2009)		
Rowlands (2004)		
Shrestha and Bhandari (2007)		

An asterisk indicates studies part of the EU’s EACH-FOR project (www.each-for.eu)

A first observation is that quantitative studies must, of necessity, simplify the many complex variables that influence migration decisions (such as access and valuation of human, social, and physical capital). Further, models may not differentiate between who is mobile and who is not, which is often a result of factors such as class, gender, age, and ethnicity. Some of these limitations may be due to the availability or comparability of data, while in others the technique itself necessitates isolation of a finite number of variables for analysis. In several instances, researchers have taken advantage of datasets obtained from national household surveys (e.g., Findley 1994; Henry et al. 2004a, b) or that were generated in other research projects, such as the Mexican Migration Project (http://mmp.opr.princeton.edu/, used by Nawrotzki et al. 2013) and long-term social development research in Nepal (Massey et al. 2010; Shrestha and Bhandari 2007). Since not all countries and jurisdictions have household survey data, use of modelling is often limited to those jurisdictions where it does exist. The environmental data used in modelling studies often appear to reflect that which is readily available, such as precipitation and average temperatures, and may not necessarily reflect the full range of potential environmental factors (or their interactions with one another) that may influence migration. For example, in tracing environmental motivations for migration out of rural Saskatchewan in the 1930s (which included small amounts of migration to the US), McLeman and Ploeger (2012) and McLeman et al. (2010) found it necessary to combine three different types of historical climate data (July–September maximum daily temperatures, May–July precipitation data, and October–December precipitation data for the preceding year), and soil quality information before reliable associations between environment and migration and local-level migration could be identified.

In some cases, even researchers looking at the same country and environmental factor but using different methods could arrive at different conclusions. In studying the migration effects of drought in Burkina Faso, Henry et al. (2004a, b) used hazard analysis models and found little evidence that drought leads to international migration (although it does affect internal migration), while agent-based models used by Kniveton et al. (2011) predict the highest international migrant flows in a dry climate scenario combined with “low demographic growth and inclusive and connected social and political governance” (p. 539). Kniveton et al. (2011) believe this discrepancy could be due to their model’s over-sensitivity to the influence of others on agent behavior, or to their own inclusion of migration out of urban centers while Henry et al. (2004b) focus on the Sahel and central regions.

As another example, studies by Massey et al. (2010) and Shrestha and Bhandari (2007) came to different conclusions about the links between environmental insecurity and international migration patterns from Nepal’s Chitwan Valley. Massey et al. (2010) found that increased time taken to collect firewood was more likely to lead to internal rather than international migration, while Shrestha and Bhandari (2007) found that an increased time taken to collect firewood contributed equally to internal and international migration. Although both utilize the same 1996 household survey data, Massey et al. (2010) additionally conducted life history interviews and land use measurements. As well, both studies use different temporal scales to define “migration” and utilize some different environmental and non-environmental variables.

Quantitative techniques prove best able to identify actual and potential population movements in broad terms and at fairly large spatial scales. By contrast, the six empirical studies that employ qualitative methods (the most common one being interviews of migrants, non-migrants, and/or key informants) appear to be most successful in identifying migrants’ motivations, the role of environmental factors within those motivations, and the interplay of environment with non-environmental factors in causality. It is, however, worth noting that qualitative studies are not foolproof in achieving such ends, and there are a number of studies that raise potential cautions with respect to interviewees’ perceptions of environmental risks, selectivity in assigning migration causality, and other methodological challenges (e.g. Barbier et al. 2009; Hunter et al. 2010; van der Geest 2010). Eleven of the studies employ a mixture of qualitative and quantitative methods, examples of the latter typically being surveys and simple statistical analyses. Given the costs and time involved, these mixed-method and qualitative studies tend to focus on migration patterns at smaller spatial scales. Targeted samples of people living in areas exposed to environmental risks, with participants recruited “snowball style” (i.e., where one informant leads researchers to others), are the most common methodological approach; randomized sampling is not typically practiced. This approach creates a risk of sampling bias, whereby all or no participants may have experienced, and/or migrated due to, environmental stress (Warner 2011). In her analysis of the methodology of the European Union-funded EACH-FOR project (of which seven articles appear in our inventory), Warner (2011) notes that the timing of research often affected the outcomes. In many of the study areas, the seasonality and nature of labor is such that composition of the pool of potential informants changes throughout the year (even the time of day when interviews are done may have an influence). A further potential bias observed by EACH-FOR researchers was that those living in areas where environmental stress had been experienced more recently or regularly were more likely to be able to speak to the effects of environmental events or stressors than those with no experience or where the experience was long in the past. In this case, quantitative studies may be better equipped to see changes in the longer term.

Five of the qualitative methods articles use poststructuralist discourse analysis with which they investigate how discourses about environmental or climatic change and migration have been constructed (Bettini 2013; Farbotko 2005; Farbotko and Lazrus 2012; McNamara 2007; McNamara and Gibson 2009). Drawing upon social justice frameworks, these analyses show that unequal relations of power in the processes of defining and framing environmental migration—by politicians, media, humanitarian NGOs, and even academics—have policy implications. Concerns are raised by these authors that the label “refugee” is often imposed on environmental migrants, thus depicting them as vulnerable, disempowered, and without agency—a representation that is inconsistent with studies showing that migration is actually an important adaptive response to environmental stress. What is more critical than raising fears about “environmental refugees” is to identify barriers to adaptation and mobility (e.g., McLeman and Smit 2006; Bardsley and Hugo 2010; Black et al. 2011). With the exceptions of Farbotko and Lazrus (2012) and McNamara and Gibson (2009), it is not clear that members of populations experiencing environmentally-related migration have actively participated in discourse analysis research, creating a useful opportunity for future studies.

## Discussion and conclusions

Several important issues emerge from our review of the empirical literature and suggest a number of considerations for future research and policymaking. The first of these relates to research methods and design. Beyond the obvious need for more empirical research on environmental influences on international migration generally, there is a clear need for more longitudinal studies. Most empirical studies currently available focus on relatively short time periods, offering only partial snapshots of human–environment interactions that unfold over many years or decades. In addition to encouraging researchers to pursue longer-duration studies, it would be beneficial if authors of already-published research could conduct periodic follow-up studies to track future developments among their study participants and communities. An ancillary benefit of longer-duration studies is that they potentially allow for greater differentiation of internal and international migration and consider the role of alternative adaptations. This is not always easy to do in short duration studies, and in reviewing the available empirical research, we have found it challenging to draw out conclusions about international environmental migration specifically. Another option for extending longitudinal reach is through studies that hindcast environmentally-related migration using historical data, such has been done by Jennings and Gray (2013) for nineteenth century Holland or McLeman and Ploeger (2012) for 1930s Saskatchewan, Canada.

We also observed that it is difficult to make general conclusions as to which types of environmental factors may be most likely to influence international migration. While research is inevitably constrained by the availability of data, there is a need to expand the range of environmental events and conditions that are examined to determine their potential influences on migration behavior. While we found a relatively large number of studies that investigated drought, this should not be interpreted as meaning that drought is the greatest or most common environmental motivator of migration; it may simply reflect that rainfall data are relatively easy to come by, or that population movements related to drought are relatively simple to recognize. We further note that existing empirical research focuses mainly on migration driven by rural environmental problems, and that urban environmental challenges such as untreated wastewater, air pollution, and land contamination are under-studied.

Consistent with recommendations made previously by Piguet (2010) and Warner (2011), our review leads us to encourage wider adoption of mixed research methods wherever possible. All methodological approaches have their strengths and limitations. The “ground truthing” of quantitative or modelling research through qualitative inquiry, for example, increases the potential that the environmental variables being used in models reflect the migration pressures as they are perceived or experienced by people on the ground, and not simply the availability of data. The studies we reviewed suggest that the environmental influence on migration may be felt through second- or third-order impacts that are not easily captured even through very sophisticated multivariate hazard modeling, a good example being Alscher’s (2011) account of the complex interactions of deforestation, soil erosion, political, and socioeconomic processes that influence migration out of rural Dominican Republic and Haiti. By the same token, qualitative case studies of migrant experiences offer detailed insights into decision-making considerations and processes at very local levels; the next challenge is determining to what extent these observations scale up (i.e., are reflective of population-wide behaviors more broadly). This question is of particular interest to policymakers, and the methodological addition of quantitative analyses is often an important next step in providing the answer.

The empirical studies suggest that context plays a tremendous role in shaping environmentally-related migration outcomes across borders. Generally, the articles reviewed here could provide further information about the environmental and especially the non-environmental context of their empirical case studies. The role of context is under-theorized, and we believe that more research is needed to understand the dynamics between environmental and non-environmental factors at a range of scales. Similarly, more detailed examination of the interactions between various non-environmental structural factors at various scales could help advance understanding of the complex structure–agency dynamics that shape migration decisions. In response to the many unknowns regarding why certain variables lead to different international migration outcomes in different settings, future studies could consider conducting comparative analyses looking at a range of scalar factors. This type of comparison could perhaps shed light on why education, for example, leads to different types of migration in different countries.

The following exploratory framework may help future studies integrate a dynamic understanding of the role of the broader environmental and non-environmental context in their research. For the environmental context, an important starting point is to grasp the local ecology of a case study and how it relates to ecologies at the regional and broader scales (including global environmental change). The specific processes of environmental change experienced by a group of people and in a given region need to be understood contextually, since their manifestation and impacts on local livelihoods will be unique.

At the same time, studies might consider how these environmental factors interact with a range of non-environmental processes at various scales. First, empirical case studies need to be better contextualized in terms of the broader political and economic structures of the nations where they are located, including a country’s level of development and its position within the broader context of economic and political globalization. A macro-historical perspective of a country’s or region’s experiences with colonial, postcolonial, and neocolonial power relations can help to explain its current circumstances—specifically its vulnerability and adaptive capacity—while also advancing more critical understandings of structure–agency dynamics on a global scale. Furthermore, taking into account the global governance of migration will illuminate what types of formal assistance are available to people affected by environmental problems (Warner 2010; McNamara 2007).

In turn, a detailed portrait of the national conjuncture, especially the political, economic, and social institutions available, will provide important information to contextualize the impacts of environmental change. While the conjuncture is often related to level of economic development, there may be significant differences between countries with similar levels of development when it comes to state programmes and policies (especially in relation to the environment), economic support and infrastructure (particularly for agriculture), and social service provision (e.g., education, health). The presence and/or absence of state support and programmes play a complex role in influencing the migration outcomes of environmental change, and therefore need to be scrutinized carefully, with one example being the differential impacts of resettlement depending on a range of micro and macro factors.

This brings us to contextual factors at finer scales. Striking in the literature reviewed here is the role of community, household, and individual context, particularly gender and class. While a number of articles illustrate the gendered and classed nature of environmental migration through their empirical findings (e.g., Afifi 2011; Alscher 2011; Dun 2011; Findley 1994; Gray 2010; Radel et al. 2010; Wrathall 2012), there is considerable room for greater analysis of the ways these markers of difference may affect how people cope with environmental change through mobility and migration. The existing research makes clear that a person’s multifaceted identity and access to social and physical capital plays a large role in granting him or her access to mobility and in adapting to environmental stress. In the cases reviewed, we saw that those who are left behind when environmental migration occurs are often women, children, and the elderly, and even with the potential benefit of remittances, it is they who are left to cope with rapidly deteriorating environmental conditions. We agree with Winkels’ (2012) observation that environmental migration poses risks not simply to the migrant, but to other members of the migrant’s social network, and with Black et al. (2013) that in many cases it is not the environmental *migrants*, but the *immobile*—those affected by environmental change who are not able to move—who are the most vulnerable. Black et al. (2013) have suggested that:

the ability to move is broadly correlated with wealth, level of capital (financial, human, social), the availability of places to move to, and fear of what would happen to property and assets left behind, so that broadly speaking, poorer people are generally less able to migrate even if they wish to do so. (p. s36)

We would go further, and suggest that in addition to access to capital, the empirical evidence suggests that future research needs to look more closely at the gendered, classed, and aged phenomenon of “environmental immobility”. In addition to asking, “Who are environmental migrants?” we as researchers also need to be asking, “Who are not environmental migrants?” The empirical findings from international environmental migration studies remind us that the ability to migrate, whether by choice or as the “environmental refugee” of popular discourse, is not universal and that power relations at multiple scales help determine access to mobility. We encourage future empirical work to investigate a wide range of socioecological factors that may lead to both mobility and immobility.

An expansion in the types of questions asked will inevitably require scholars of international environmental migration to engage head-on with structure versus agency debates that already occur in other areas of migration research (see Bakewell 2010; King 2012). This is not to be feared, but embraced, as we believe that the empirical research reviewed here has great potential to contribute to wider theoretical discussions. For example, as described above, while some articles in our inventory include participants describing their motivations for migration as being economic and/or political, even when environmental problems were quite prominent, the authors typically do not reflect on how the perspectives of the study participants may relate to structure and agency. We believe this is an oversight, especially in qualitative studies where such rich empirical findings are possible. By examining the positionality of individuals and households experiencing environmental change in relation to multi-scalar power relations, more light can be shed on the complex interplay between individual capacities to make choices on the one hand, and the broader environmental, social, economic, and political constraints on these decisions on the other. Active and critical engagement with the role of agency and structure in environmental migration is important not only for conceptual advancement, but also for developing policies and programs that are relevant, feasible, and more socially just. A key step toward this goal is to include migrants and community members as partners in research development and design, and not simply as subjects of research. In doing so, researchers may wish to draw upon methodological tools developed in feminist, human rights, and mobilities scholarship, where unequal power relations are a regular topic of research.

The studies we reviewed show that what is described as environmental migration includes a wide range of migratory paths and opportunities, from short term to permanent and short distance to long distance, and relatively little of it is international or permanent. Instead, the evidence shows environmental migration to be a continuum of possibilities that individual migrants and households pursue depending on their particular circumstances and the broader context in which they are located (see also McLeman 2010). In its infancy, environmental migration research often presented a simple dichotomy of environment versus society, attempting to understand the role of “environmental” factors separately from other factors motivating migration (see Gemenne 2011). In the studies we reviewed, we see an evolution toward greater treatment of environment and society as being fundamentally entwined. Although the separation of environmental and non-environmental variables will continue to be important for analytical purposes, we encourage scholars to push continually toward dynamic models (conceptual and empirical) where human-environment interactions are intrinsically embedded in one another.

Summing up, our review suggests that, at least for the present, international migration for obvious environmental reasons is not occurring in vast numbers. There is evidence that second- and third-order impacts of environmental events and conditions also influence migration decisions, but there is insufficient evidence to offer an opinion as to how much larger the global number of international environmental migrants would be if these were taken into account. No definite conclusions can yet be drawn with respect to how international migration trends or patterns respond to specific environmental factors, or to specific socioeconomic drivers, individual circumstances, or combinations thereof; at this point, all that can be said with confidence is that there are many possibilities. The potential barriers and restrictions to mobility and international migration are many and include less-studied ones such as gender and social class. Particular regions are considerably under-represented in the English language literature—especially South America and the Middle East—and little research has been done on the dynamics of environmental migration in receiving countries. Research emphasis to date has been on the environmental effects of migration from and within rural (often subsistence) populations, with little attention having been given to urban settings and urban environmental challenges. In short, the avenues and opportunities for future research are considerable, and there is much yet to be done to create a deeper understanding of the role international migration may yet play in people’s abilities to cope with environmental change.

## Electronic supplementary material

Below is the link to the electronic supplementary material.

Supplementary material 1 (DOC 163 kb)

Supplementary material 2 (DOC 27 kb)
