# Supplementary material for: Empirical research on international environmental migration: a systematic review
Source: Popul Environ. 2014 Feb 22;36(1):111–35. doi: 10.1007/s11111-014-0210-7 (PMC4131126; doi:10.1007/s11111-014-0210-7)
Supplement: Supplementary file 1 — Supplementary material 1 (DOC 163 kb) [file 11111_2014_210_MOESM1_ESM.doc]

| **Environmental International Migration Questionnaire** | |  |  |
| --- | --- | --- | --- |
|  |  |  |  |
| **ARTICLE INVENTORY** | ENTER DATA IN CELLS BELOW |  |  |
| Lead author family name: |  |  |  |
| Year published: |  |  |  |
| Article title: |  |  |  |
| Journal title: |  |  |  |
| Journal field code (see 'codes' sheet): |  |  |  |
| First author affiliation: |  |  |  |
|  |  |  |  |
| **QUANTITATIVE MEASURES** |  | ENTER 1 IF YES, 0 IF NO  (ONLY ONE CELL CAN HAVE ENTRY = 1) | |
| What is the primary focus of the article? | Internal migration |  |  |
|  | International migration |  |  |
|  | Internal + international migration |  |  |
|  |  | ENTER 1 IF YES, 0 IF NO (ONLY ONE CELL CAN HAVE ENTRY = 1) | |
| Spatial scale of the migration discussed: | Local/community |  |  |
|  | Regional (within a country) |  |  |
|  | National |  |  |
|  | International (between countries) |  |  |
|  | World Region (within a continent) |  |  |
|  | Global (many regions, many countries, complex flows) |  |  |
|  | Multiple scales |  |  |
|  |  | ENTER 1 IF YES, 0 IF NO (ONLY ONE CELL CAN HAVE ENTRY = 1) | |
| Which of following time scales is the | Historical (pre - 1900) |  |  |
| author attempting to understand the | Recent past (1900 - present) |  |  |
| behavior of the system? | Current/ongoing processes |  |  |
|  | Future behavior |  |  |
|  | Past + present + future |  |  |
|  | Specific time period (specify) |  |  |
|  |  | Enter 1 if yes, 0 if no  (enter as many 1's as apply) | |
| What world region does the article examine? | Africa |  |  |
|  | Latin America and the Caribbean |  |  |
|  | Northern America |  |  |
|  | Asia |  |  |
|  | Europe |  |  |
|  | Oceania |  |  |
|  |  | ENTER 1 IF YES, 0 IF NO  (ONLY ONE CELL CAN HAVE ENTRY = 1) | |
| Are the findings based on empirical or | Empirical |  |  |
| non-empirical evidence: | Non-empirical |  |  |
|  |  | ENTER 1 IF YES, 0 IF NO  (ONLY ONE CELL CAN HAVE ENTRY = 1) | |
| If **empirically** based, what was the | Qualitative |  |  |
| methodology used? | Quantitative |  |  |
|  | Mixed |  |  |
|  |  | Enter 1 if yes, 0 if no  (enter as many 1's as apply) | |
| If **qualitative**, which of the following | Questionnaire or survey |  |  |
| methods were used? | Interview |  |  |
|  | Case study |  |  |
|  | Observation |  |  |
|  | Collaborative action research |  |  |
|  | Other |  |  |
|  |  | Enter 1 if yes, 0 if no  (enter as many 1's as apply) | |
| If **quantitative**, which of the following | Spatial analysis (GIS) |  |  |
| methods were used? | Remote sensing |  |  |
|  | Demographic analysis |  |  |
|  | Questionnaire or survey |  |  |
|  | Regression analysis (simple or multiple) |  |  |
|  | Modelling |  |  |
|  |  | ENTER 1 IF YES, 0 IF NO  (ONLY ONE CELL CAN HAVE ENTRY = 1) | |
| If **non-empirically** based, what is the | Literature review |  |  |
| purpose of the article? | Policy discussion |  |  |
|  | Legal discussion |  |  |
|  | Theoretical or conceptual development |  |  |
|  | Framework creation |  |  |
|  |  | Enter 1 if yes, 0 if no | |
| Does the article discuss lack of data or issues about collecting and accessing data? | |  |  |
|  |  | Enter 1 if yes, 0 if no | |
| Does the article discuss methodological issues? |  |  |  |
|  |  | Enter 1 if yes, 0 if no | |
| Does the article discuss the reliability of the data? | |  |  |
|  |  | Enter 1 if yes, 0 if no | |
| Does the article further the discussion of how research methods could be improved? | |  |  |
|  |  | Enter 1 if yes, 0 if no  (enter as many 1's as apply) | |
| What environmental factors does the article | Desertification |  |  |
| explore as determinants of migration? | Drought |  |  |
|  | Food security |  |  |
|  | Land degradation |  |  |
|  | Resource scarcity |  |  |
|  | Salination |  |  |
|  | Sea level rise |  |  |
|  | Water Resources |  |  |
|  | Fire |  |  |
|  | Flooding |  |  |
|  | Landslides |  |  |
|  | Natural disasters |  |  |
|  | Rainfall |  |  |
|  | General climate change (non-specific) |  |  |
|  |  | ENTER 1 IF YES, 0 IF NO  (ONLY ONE CELL CAN HAVE ENTRY = 1) | |
| Are these factors primarily rapid or | Rapid-onset |  |  |
| slow onset? | Slow-onset |  |  |
|  |  | ENTER 1 IF YES, 0 IF NO  (ONLY ONE CELL CAN HAVE ENTRY = 1) | |
| Is the migration in question classified as forced | Forced |  |  |
| or voluntary by the author? | Voluntary |  |  |
|  | Both |  |  |
|  | Grey area |  |  |
|  |  | ENTER 1 IF YES, 0 IF NO  (ONLY ONE CELL CAN HAVE ENTRY = 1) | |
| Is there a discussion of the difficulties in classifying the migration as forced or voluntary? | |  |  |
|  |  | Enter 1 if yes, 0 if no  (enter as many 1's as apply) | |
| What types of migrants are discussed? | Environmental refugees |  |  |
|  | Environmental migrants |  |  |
|  | Economic migrants (including skilled migrants) |  |  |
|  | Refugees |  |  |
|  | Asylum seekers |  |  |
|  | Illegal, undocumented or unauthorised migrants |  |  |
|  | Temporary migrants |  |  |
|  |  | ENTER 1 IF YES, 0 IF NO  (ONLY ONE CELL CAN HAVE ENTRY = 1) | |
| The articles recommendations/discussion | Refugee law |  |  |
| focus on: | Climate change adaptation |  |  |
|  | Human health |  |  |
|  | Economic policy |  |  |
|  | International migration law |  |  |
|  | Strategies for the resettlement of populations |  |  |
|  | Regulation of migration |  |  |
|  | Security |  |  |
|  | Other |  |  |
|  |  | Enter 1 if yes, 0 if no  (enter as many 1's as apply) | |
| What actors are discussed in the article as the | Individuals |  |  |
| responsible/affected/involved ? | Nation States |  |  |
|  | NGO's (humanitarian, international development) |  |  |
|  | International bodies (UN) |  |  |
|  | Ethnic communities |  |  |
|  |  | ENTER 1 IF YES, 0 IF NO  (ONLY ONE CELL CAN HAVE ENTRY = 1) | |
| Which individuals are chosen as the | Individuals |  |  |
| subject of the study? | Families |  |  |
|  | Communities |  |  |
|  | Mass migration or mass displacement |  |  |
|  | Other (Describe) |  |  |
|  |  | Enter 1 if yes, 0 if no  (enter as many 1's as apply) | |
| The article discusses issues of: | Gender |  |  |
|  | Race or ethnicity |  |  |
|  | Class |  |  |
|  |  | Enter 1 if yes, 0 if no  (enter as many 1's as apply) | |
| Does the article touch on these types | rural-urban |  |  |
| of geographic migration? | urban-rural |  |  |
|  | rural-rural |  |  |
|  | inter-urban |  |  |
|  |  | Enter 1 if yes, 0 if no  (enter as many 1's as apply) | |
| Does the article touch on: | first-time migration |  |  |
|  | return migration |  |  |
|  | repeat migration |  |  |
|  | chain migration |  |  |
|  | transnationalism |  |  |
|  |  | Enter 1 if yes, 0 if no  (enter as many 1's as apply) | |
| Does the article deal with issues of definition of: | Refugee |  |  |
|  | Environmental Refugee |  |  |
|  | Environmental Migrant |  |  |
|  |  | Enter 1 if yes, 0 if no | |
| Does the paper challenge or address the structure vs. agency debate? | |  |  |
|  |  | Enter 1 if yes, 0 if no  (enter as many 1's as apply) | |
| The focus of the paper is: | Structure |  |  |
|  | Agency |  |  |
|  |  | Enter 1 if yes, 0 if no  (enter as many 1's as apply) | |
| Is this project part of another project: | EACH-For |  |  |
|  | Foresight |  |  |
|  |  |  |  |
| **QUALITATIVE MEASURES** |  |  |  |
| What are the main arguments | Each box is for separate ideas. |  |  |
| discussed in the article? | Write in short phrases with key words to be used |  |  |
|  | later when coding the responses. |  |  |
|  |  |  |  |
|  |  |  |  |
|  |  |  |  |
| What future research is recommended by the article? | |  |  |
|  |  |  |  |
| Is there any important information from the article in addition to what is mentioned above? | |  |  |
|  |  |  |  |
| Is there anything mentioned in the article which seems extremely important or noteworthy? | |  |  |
|  |  |  |  |
| Is there anything that you would like to recall for later? | |  |  |
|  |  |  |  |

**Codes**

| **Field Code** | **Journal field** |
| --- | --- |
| 1 | Climate Change |
| 2 | Demography/migration/population studies |
| 3 | Ecology/Biology (excluding agricultural sciences) |
| 4 | Economics |
| 5 | Environmental Science (physical drivers of migration) |
| 6 | Health and Environment |
| 7 | International Development |
| 8 | Law and Policy |
| 9 | Political science |
| 10 | Social Science |
| 0 | Other (add remark) |
