# Supplementary material for: Empirical research on international environmental migration: a systematic review
Source: Popul Environ. 2014 Feb 22;36(1):111–35. doi: 10.1007/s11111-014-0210-7 (PMC4131126; doi:10.1007/s11111-014-0210-7)
Supplement: Supplementary file 2 — Supplementary material 2 (DOC 27 kb) [file 11111_2014_210_MOESM2_ESM.doc]

**Inventory of Articles Reviewed**

Afifi, T. (2011). Economic or environmental migration? The push factors in Niger. *International Migration*, 49(S1), e95-e124.

Alscher, S. (2011). Environmental degradation and migration on Hispaniola Island. *International Migration*, 49(S1), e164-e188.

Bettini, G. (2013). Climate barbarians at the gate? A critique of apocalyptic narratives on ‘climate refugees’. *Geoforum*, 45, 63-72.

Dun, O. (2011). Migration and displacement triggered by floods in the Mekong delta. *International Migration,* 49(S1), e200-e223.

Farbotko, C. (2005). Tuvalu and climate change: Constructions of environmental displacement in the Sydney Morning Herald. *Geografiska Annaler*, 87B, 279- 293.

Farbotko, C. and Lazrus, (2012). The first climate refugees? Contesting global narratives of climate change in Tuvalu. *Global Environmental Change*, 22, 382-390.

Feng, S., Krueger, A.B., Oppenheimer, M. (2010). Linkages among climate change, crop yields and Mexico-US border migration. *PNAS*, 107(32), 14257-14262.

Findley, S. E. (1994). Does drought increase migration? A study of migration from rural Mali during the 1983-1985 drought. *International Migration Review*, 28(3), 539-553.

Gila, O.A., Zaratiegui, A.U. & Lopes de Maturana Dieguez, V. (2011). Western Sahara: Migration, exile and environment. *International Migration*, 49(S1), e146- e163.

Gray, C.L. (2009). Environment, land, and rural out-migration in the southern Ecuadorian Andes. *World Development*, 37(2), 457-468.

Gray, C. L. (2010). Gender, natural capital, and migration in the southern Ecuadorian Andes. *Environment and Planning* A, 42, 678-696.

Henry, S., Piche, V., Ouedraogo, Lambin, E.F. (2004). Descriptive analysis of the individual migratory pathways according to environmental typologies. *Population and Environment*, 25(5), 397-422.

Henry, S., Schoumaker, B. and Beauchemin C. (2004). The impact of rainfall on the first out-migration: A multi-level event-history analysis in Burkina Faso. *Population and Environment*, 25(5), 423-460.

Kniveton, D., Smith, C. & Wood, S. (2011). Agent-based model simulations of future changes in migration flows for Burkina Faso. *Global Environmental Change*, 21S, s34-s40.

Marchiori, L., Maystadt, J-F., Schumacher, I. (2012). The impact of weather anomalies on migration in sub-Saharan Africa. *Journal of Environmental Economics and Management*, 63, 355-374.

Marchiori, L. & Schumacher, I. (2011). When nature rebels: international migration, climate change, and inequality. *Journal of Population Economics*, 24, 569-600.

Massey, D.S., Axinn, W.G., Ghimire, D.J. Environmental change and out-migration: Evidence from Nepal. *Population and Environment*. 32, 109-136.

McNamara, K.E. (2007). Conceptualizing discourses on environmental refugees at the United Nations. *Population and Environment*, 29, 12-24.

McNamara, K.E. & Gibson, C. (2009). We do not want to leave our land: Pacific ambassadors at the United Nations resist the category of ‘climate refugees’. *Geoforum*, 40, 475-483.

Mortreux, C. and Barnett, J. (2009).Climate change, migration and adaptation in Funafuti, Tuvalu. *Global Environmental Change*, 19, 105-112.

Nawrotzki, R.J., Riosmena, F. and Hunter, L.M. Do rainfall deficits predict U.S.-bound migration from rural Mexico? Evidence from the Mexican census. *Populations Research and Policy Review*, 32, 129-158.

Radel, C., Schmook, B. and McCandless, S. (2010). Environment, transnational labor migration, and gender: case studies from southern Yucatan, Mexico and Vermont, USA. *Population and Environment*, 32, 177-197.

Reuveny, R. and Moore, W.H. (2009). Does environmental degradation influence migration? Emigration to developed countries in the late 1980s and 1990s. *Social Science Quarterly*, 90(3), 461-479.

Rowlands, D. (2004). The effects of poverty, environmental degradation, and gender conditions on south-to-north migration. *Canadian Journal of Development Studies*, XXV(4), 555-572.

Shen, S. & Binns, T. (2012). Pathways, motivations and challenges: Contemporary Tuvaluan migration to New Zealand. *GeoJournal*, 77, 63-82.

Shen, S. & Gemenne, F. (2011). Contrasted views on environmental change and migration: The case of Tuvaluan migration to New Zealand. *International Migration*, 49(S1), e224-e242.

Shrestha, S.S. & Bhandari, P. (2007). Environmental security and labor migration in Nepal. *Population and Environment*, 29, 25-38.

Sunil, T.S., Rojas, V. & Bradley, D.E. (2007). United States’ international retirement migration: the reasons for retiring to the environs of Lake Chapala, Mexico. *Ageing & Society*, 27, 489-510.

Warner, K. (2010). Global environmental change and migration: Governance challenges. *Global Environmental Change*, 20, 402-413.

Warner, K., Hamza, M., Oliver-Smith, A., Renaud, F and Julca, A. (2010). Climate change, environmental degradation and migration. *Natural Hazards*, 55, 689-715.

Wrathall, D.J. (2012). Migration amidst social-ecological regime shift: The search for stability in Garifuna villages of northern Honduras. *Human Ecology*, 40, 583-596.
